# Evaluation of panoramic images in the assessment of mandibular second molar impaction

**DOI:** 10.4317/jced.62127

**Published:** 2024-11-01

**Authors:** Kathleen R. McKeon, Robert R. Mortimer, John M. Burnheimer

**Affiliations:** 1 Former resident, Department of Orthodontics and Dentofacial Orthopedics, School of Dental Medicine, University of Pittsburgh, Pittsburgh, PA; 2 Clinical Assistant Professor, Department of Orthodontics and Dentofacial Orthopedics, School of Dental Medicine, University of Pittsburgh, Pittsburgh, PA; 3 Associate Professor, Advanced Education Program in Orthodontics and Dentofacial Orthopedics, Seton Hill University, Greensburg, PA

## Abstract

**Background:**

To examine the relationship of various mandibular skeletal features determined from assessment of a panoramic image to the impaction of mandibular second molars in children with late mixed to early permanent dentition.

**Material and Methods:**

Thirty-six panoramic radiographs were collected from two private orthodontic offices of consecutively screened patients in the late mixed to early permanent dentition. Gonial angle, space ratio, ramus ratio and occlusal plane to posterior ramus ratio were analyzed for any significant relationship with mandibular second molar impaction. Measurements were made on each of the orthopantomograms and compared between the impaction and non-impaction groups.

**Results:**

The overall sample consisted of 36 patients between the ages of 11 to 14 years with a mean age of 12.5 years and included 21 females and 15 males. Eighteen were identified as having impactions and were designated to the IMP group, while eighteen without impactions were designated to the NON group. There was a significant difference in the tooth size to space available ratio between groups with the second molar accounting for 126% of the space available in the IMP group and about 80% of the space available in the NON group (*P*<0.001). Additionally, a significant difference was found in occlusal plane to posterior ramus angle between the two groups, with the IMP group averaging 73.93º, and the NON group averaging 66.39º (*P*=0.002).

**Conclusions:**

The mandibular second molars of patients presenting with impactions occupy more of the available space and exhibit a greater occlusal plane to posterior ramus angle.

** Key words:**Impaction, gonial angle, second molar.

## Introduction

In general, second molar impaction is a rare phenomenon with a prevalence between 0.6% 

and 3% (1,2). In an epidemiological study with a population sample of 4063, Aitasala *et al*. (3) found that 14.1% of the population had one or more impactions, but that less than 1% of these impactions were first and second molars. In a Swedish population, Varpio (2) found a prevalence of impaction of second molars to be 0.15%, while Johnsen (4) found a prevalence of 0.3% in an American population. More recent studies have found the prevalence of second molar impaction ranging from 0.2% to 1.36% in a predominately white population (5,6). In a Taiwanese population, Fu *et al*. (7) found a 0.65% impaction prevalence whereas Cho *et al*. (8) found a 1% prevalence of impacted second molars in a Chinese population.

A few theories of causality, include lower arch crowding, genetics, and the use of appliances that preserve E-space, such as lower lingual holding arches and lip bumpers, have been suggested (9-12). In a cross-sectional study performed by Evans, it was shown that there was an increase in second molar impaction in orthodontic patients over a 10-year period, and that lower arch crowding was the most consistent finding within the impacted group (9). While there have been a few proposed theories, the etiology of second molar impaction remains unclear. A retrospective study by Ferro collected data on orthodontic patients with lower crowding (12). The experimental group in this study was treated with lip bumpers to prevent mesial drifting of the permanent first molar, and this result suggested that the lip bumper may be a potential cause for second molar impaction (12). Another study, performed by Sonis *et al*. (11) looked at the preservation of E-space in two hundred patients and found that second molar impaction was increased in those that received space maintainers when compared to those that did not, again implicating the prevention of mesial drift in second molar impaction cases. A study performed by Shapira *et al*. (10) suggests a genetic component in second molar impactions, with a greater prevalence in a Chinese-American population when compared to a commensurate Israeli population. Other studies have shown a connection between vertical condylar growth and third molar impaction (13-6), suggesting that an increased vertical condylar growth pattern leads to a decrease in gonial angle (GA), and therefore a smaller space between the mandibular second molar and the anterior border of the ramus (13,14).

Failure of a second molar to erupt is typically an asymptomatic pathology and is frequently an incidental finding (17). Impaction has been defined in multiple ways including a tooth that has an abnormal contact with a tooth in the same arch (9), a tooth which fails to complete eruption to occlusal height during the time it should have emerged or a tooth which remains below the cementoenamel junction of the adjacent mandibular first molar (6,11). Many studies consider a tooth to be impacted by following the Raghoebar *et al*. (18) definition, which states that a tooth is impacted when there has been a cessation of eruption caused by a clinically or radiographically detecTable physical barrier in the eruption path or due to an abnormal position of the tooth.

While GA has been traditionally measured using cephalometric radiographs, Mattila *et al*. (19) did a study using panoramic and cephalometric radiographs from 601 patients that showed that GA measurements were similar using both radiographic methods. That study, and one by Radhakrishnan *et al*. (20), demonstrated that the GA can be measured from a panoramic radiograph with the same degree of accuracy as from a cephalometric radiograph. Other authors have stated that horizontal measurements are particularly unreliable. However, Zuniga (21) found that even though there is greater variability in the horizontal measurements, the reliability is high to very high.

While multiple studies have been done on impactions including a review by Neychev *et al*. (22), current research lacks investigation into the effect of mandibular skeletal features on second molar impaction when viewed on a panoramic radiograph. The aim of this study will be to examine the relationship of various mandibular skeletal features determined from assessment of a panoramic image to the impaction of mandibular second molars in children with late mixed to early permanent dentition.

## Material and Methods

De-identified panoramic radiographs were sequentially collected from two private orthodontic offices. After a clinical examination and parental informed consent, panoramic radiographs were obtained for each patient to complete the initial orthodontic records appointment. The patient was positioned with the incisors in the groove in the bite block, head straight, midsagittal plane perpendicular to the floor and Frankfort plane parallel with the floor. Inclusion criteria for the study included consecutively examined patients of ages 11-14 with panoramic radiographs. The panoramic radiographs were evaluated for the presence of impacted second molars, utilizing the definitions of a previously published study by Raghoebar *et al*., which states the cessation of eruption caused by a clinically or radiographically detectable physical barrier in the eruption path or due to an abnormal position of the tooth (18). Exclusion criteria included patients with craniofacial anomalies/syndromes, previous orthodontic treatment, or any missing mandibular teeth (excluding third molars). Mean age of the subjects was 12.5 years with a range of 11 to 14 years and in the late mixed to early permanent dentition. Those with impactions were designated as the impaction group (IMP), while those without impaction were designated as the non-impaction group (NON).

**Figure 1:**
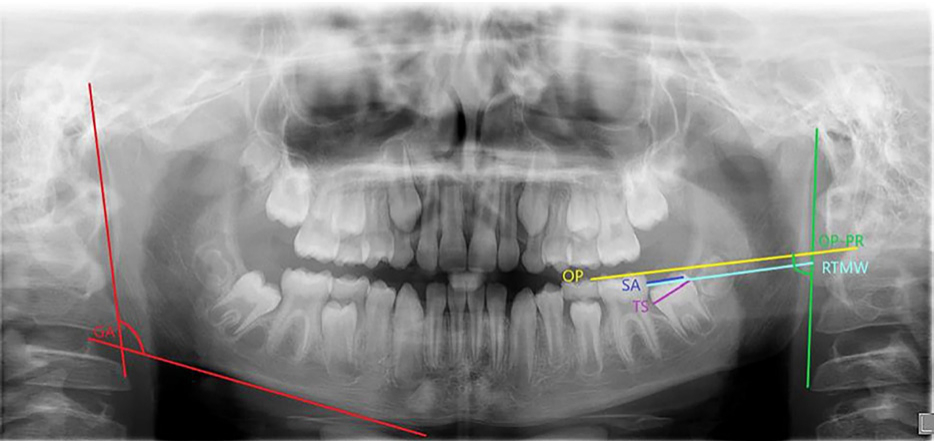
Construction of angular and linear measurements. GA (red) is the gonial angle. OP (yellow) is the line demarking the occlusal plane. SA (dark blue) is space available (distance from distal height of contour of the mandibular first molar to the anterior border of the ramus). TS (purple) is tooth size (distance from mesial to distal height of contour. RTMW (light blue) is ramus to molar width (distance from distal height of contour of mandibular first molar to the posterior border of the ramus). OP-PR is the occlusal plane to posterior ramus angle (green arc).

The panoramic images were magnified to twice normal size to aid in utilization of the software. GA measurement was taken by measuring the angle between a line drawn tangent to the posterior border of the ramus and a second line tangent to the lower border of the mandible. The occlusal plane (OP) was then established by drawing a line tangent to the cusp tips of the mandibular first molar. The measurement for space available (SA) was made by drawing a line from the distal height of contour of the first molar to the anterior border of the ramus, parallel to the occlusal plane. Tooth size (TS) was measured from the mesial height of contour to the distal height of contour of the mandibular second molar. Ramus to molar width (RTMW) was measured from the distal height of contour of the mandibular first molar to the posterior border of the ramus, parallel to the occlusal plane. OP-PR was the angle between the occlusal plane and the line tangent to the posterior border of the ramus (Fig. 1).

After designation into IMP and NON groups, measurements were made to determine GA, tooth size, space available for mandibular second molars, ramus to molar width, and occlusal plane to posterior ramus angle (OP-PR). This was done using the simple ruler and protractor measuring tool in MiPACS software (MiPACS Dental Enterprise Viewer, Medicor Imaging, Charlotte, NC). Because these measurements were taken on panoramic radiographs from 2 different offices and to account for potential magnification errors, ratios were utilized to account for differing magnifications of the images. Linear measurements were assessed to the nearest 0.1 mm and converted into ratios (TS: SA and TS: RTMW). The measurements were taken by two separate investigators and a random sample of ten was re-measured 6 weeks later to ensure intra-rater reliability.

Due to the rare occurrence of second molar impaction, a power analysis was performed. Utilizing a power of 80%, alpha of 0.05, effect size and standard deviation of 5° for the GA, the sample size was calculated to be 18 patients per group. A chi square test was done to determine the difference between the sex in the IMP and NON groups. As assumptions of normal distribution were not provided, Mann-Whitney U tests were done to determine the differences between age, GA, TS: SA ratio, TS: RTMW ratio, and OP-PR in the IMP and NON groups. Intra- and inter-rater reliabilities were assessed. Intra-rater reliability was 0.984 and 0.862, indicating good intra-rater reliability. Inter-rater reliability was calculated to be 0.962. Investigators were unable to be blinded as to which patients were assigned to each group, due to the nature of the investigation. All statistical analysis was done using Stata/SE 15.1 software.

## Results

Eighteen subjects were selected for each group (IMP and NON), comprised of 15 males and 21 females. Seven patients presented with bilaterally impacted second molars, while 5 patients presented with right sided impactions only, and 6 patients presented with left sided impactions only. In regard to the sex of the patient, there was no statistically significant difference between the IMP and NON groups ( Table 1). The overall average age of the subjects was 12.6 years old, ranging from 11-14 years. Mean age for the IMP group was 12.43 years old and 12.76 years old for the NON group, indicating no difference between the age groups. Similarly, there were no difference in the GA between the IMP and NON groups. The average TS: SA ratio in this study was 0.97, with the IMP group at 1.26, indicating space deficiency, and 0.80 for the NON group, indicating an excess of space. Average TS: RTMW ratio in the study was 0.26, with the IMP group at 0.28, and 0.24 for the NON group. Finally, the average OP-PR in this study was 69.41°, with IMP group measuring 73.93°, and the NON group 66.39° ( Table 2).

As not all patients had bilateral impactions, the data was split into right and left sides, and these results are listed in  Table 3. There was no difference between right and left GA, however, statistically significant differences between IMP and NON groups were found for average TS: SA ratio, TS: SA ratio on the right and left sides, average TS: RTMW ratio, TS: RTMW ratio on the right and left sides, average OP-PR, and OP-PR on the right and left sides ( Table 2, Table 3).

## Discussion

The results of this study indicate that there was no difference in GA between the IMP and NON groups in this age range. This finding contrasts with that of Vedtofte *et al*., who found that a smaller GA was associated with the impaction of mandibular second molars (1). This difference in GA may be attributable to a few factors. First, the average age was nearly 3 years older than the 

present study, allowing for continued mandibular growth and remodeling. Second, radiographs were taken after the majority of patients had completed orthodontic treatment, whereas this study focused on pre-treatment radiographs. Finally, a marginally smaller sample was enrolled in that study.

A statistically significant difference was found in the ratio comparing the size of the mandibular second molar to the space available. The ratio was larger in the IMP group when compared with the NON group. A larger ratio indicates that the mandibular second molar is taking up a larger portion of the space available. In this study, the IMP group tooth size was 26% greater than the available space, while in the NON group the tooth size was 20% less than the space available, a clinically significant difference. This finding corroborates earlier studies (23), confirming that there is less space available in cases with impacted teeth.

Findings for the TS: RTMW ratio were similar to those found for the TS: SA ratio. There was a statistically significant difference between the two groups, with a larger average ratio in the IMP group (0.28), compared with the average ratio in the NON group (0.24). Again, a larger ratio indicates that the mandibular second molar is taking up a larger portion of the distance from the distal of the mandibular first molar to the posterior border of the ramus. In this case, for the IMP group, the mandibular second molar accounted for 27.86% of the space from the distal height of contour of the first molar to the posterior border of the ramus, while in the NON group, the mandibular second molar accounted for 24.19% of the space. This may indicate a larger ramal thickness in patients with second molar impactions when compared with patients without second molar impaction. While this is a statistically significant finding, this difference may not be clinically significant.

The occlusal plane to posterior ramus angle (OP-PR) measurements showed a significant difference between IMP and NON groups. On average, the OP-PR in patients with mandibular second molar impactions was 73.93º, while the average was 66.39º in non-impacted patients. This implies that patients with a flatter or more distally inclined occlusal plane are more likely to have an impacted second molar than a patient with a steeper or more mesially tipped occlusal plane, as may be seen in open bites. With an average difference of about 7° between the IMP and NON groups, this finding is clinically significant. Using this information as a diagnostic tool may assist the clinician in earlier diagnosis of second molar impaction, and therefore lead to earlier intervention.

Previous studies have shown that high or mesial angulation of the mandibular second molar increased the likelihood of impaction, but have not discussed the role of the angulation of the first molar in second molar impaction cases (1,9,11,23). This study showed a flatter or more distally tipped occlusal plane when compared with the posterior border of the ramus results in a higher likelihood of impaction. This would indicate an upright or even distal angulation of the mandibular first molar, which would lead to limited space for mandibular second molar, thereby leading to mandibular second molar impaction. In addition to indicating an upright or distal angulation of the mandibular first molar, a flatter occlusal plane often indicates a deep bite, suggesting that patients with deeper bites are more likely to have mandibular second molar impaction.

Based on the findings of this study, when treatment planning for patients with a large TS: SA ratio, a large TS: RTMW ratio, or a large OP-PR angle, the clinician should consider the possibility of an increased chance for mandibular second molar impaction when weighing the risks and benefits of lip bumper and other preservation of E-space treatments. With those patients, allowing natural mesial drift of the mandibular first permanent molar would be suggested. In cases with crowding that are on the borderline of extraction vs. non-extraction treatment, the findings from this study may be useful to the clinician when developing a comprehensive treatment plan for this age group.

Certainly, this research has several limitations. First, there were no clinical measurements directly from the patients, including amount of crowding or size of the first molars, which could have been used to account for magnification error between the different radiographs. Second, the study population consisted of patients from two suburban private orthodontic practices, which may limit the generalizability of the findings. Third, additional studies are recommended to more clearly delineate possible causative factors of second molar impaction.

## Conclusions

Based on the results of this study, the following conclusions can be made.

1. In this age group, it is recommended that patients with an occlusal plane to posterior ramus angle of greater than 73° receive more frequent monitoring of the eruption of mandibular second molars.

2. The mesio-distal dimension of impacted mandibular second molars exceed the space available.

3. Mandibular space savings appliances may be contraindicated in those patients with an OP-RA greater that 73°.

## Figures and Tables

**Table 1:** Comparison of impacted and non-impacted groups in relation to sex.

Sex comparison				
	Impacted	Non Impacted	Total	P value
Male	7	8	15	0.739*
Female	11	10	21	
Total	18	18	36	

* Chi-square test

**Table 2:** Comparison of average measurements between impacted and non-impacted groups.

	Over all Mean	SD	Mean Impacted	SD	Mean Non-Impacted	SD	P Value
Age, y	12.60	1.15	12.43	1.01	12.76	0.73	0.356
Gonial Angle º	22.75	6.61	121.98	4.88	123.52	8.10	0.468
Space Ratio	0.97	0.27	1.26	0.27	0.80	0.12	<0.001*
Ramus Ratio	0.26	0.03	0.28	0.03	0.24	0.02	0.002*
OP-PR Angleº	69.41	6.54	73.93	7.62	66.39	4.24	0.002*

* Indicates a statistically significant difference, with a *p* value of <0.05 Mann-Whitney U test SD = standard deviation y = years ° = degrees

**Table 3:** Comparison of separate right and left side measurements between impacted and non-impacted groups.

	Mean	SD	Impacted (R) n=12 (L) n=13	SD	Non Impacted (R) n=18 (L) n=18	SD	P value
Gonial Angle (R)º	122.25	6.61	120.89	5.07	122.98	8.15	0.540
Gonial Angle (L)º	123.31	7.09	123.55	6.55	123.07	7.63	0.434
Space Ratio (R)	0.97	0.28	1.26	0.24	0.81	0.12	<0.001*
Space Ratio (L)	0.96	0.31	1.26	0.25	0.79	0.13	<0.001*
Ramus Ratio (R)	0.25	0.03	0.28	0.03	0.24	0.03	0.020
Ramus Ratio (L)	0.26	0.03	0.28	0.03	0.24	0.03	<0.001*
OP-PR (R)º	69.65	6.84	74.12	7.69	67.50	5.99	0.039*
OP-PR (L)º	69.17	7.39	73.77	6.84	66.41	7.94	<0.001*

* Indicates a statistically significant difference, with *p* value of <0.05 Mann-Whitney U test SD = standard deviation ° = degrees n = number

## Data Availability

The datasets used and/or analyzed during the current study are available from the corresponding author.
